# The Application of Low-fidelity Chest Tube Insertion Using Remote Telesimulation in Training Healthcare Professionals

**DOI:** 10.7759/cureus.6273

**Published:** 2019-12-02

**Authors:** Chantae Garland, Jaime A Wilson, Michael H Parsons, Adam Dubrowski

**Affiliations:** 1 Medicine, Memorial University of Newfoundland, St. John's, CAN; 2 Emergency Medicine, Memorial University of Newfoundland, St. John's, CAN; 3 Health Sciences, Ontario Tech University, Oshawa, CAN

**Keywords:** chest tube insertion, simulation-based education, tele-simulation, emergency medicine, tension pneumothorax, rural healthcare, three-dimensional printing

## Abstract

Healthcare professionals practicing in rural, remote, or resource-restricted areas have little opportunity to practice “high stakes low-frequency” clinical procedures, despite having higher rates of injury-related death than city inhabitants. Availability of clinical skills instructors, the expense of practicing skills, lack of educational sessions, and distance to simulation centres can be a barrier to teaching and skill maintenance, particularly in rural settings. Telesimulation has the potential to overcome these challenges using audio-visual technology to connect rural learners with instructors in simulation centres. Using low-fidelity simulation models allows learners to acquire clinical skills through hands-on practice without risk or fear of harming real patients. Although not as realistic as high-fidelity models, the low-fidelity three-dimensional (3D) printed model for chest tube insertion is cost-effective and easy to set up and use and is a valid tool for teaching the clinical procedure. The purpose of this technical report was to describe the application of low-cost telesimulation to facilitate teaching chest tube insertion to medical students, emergency medicine residents, and doctors working in remote and rural environments.

## Introduction

Chest tube insertion is a vital, if infrequent, procedure for emergency medicine physicians. It is typically performed under high-stress trauma situations but can be utilized in other resuscitation situations such as drainage of pleural effusion or empyema. It is important for physicians, particularly those who work in rural and remote areas where there may not be much backup, to be proficient in the procedure. Simulation-based learners have the opportunity to acquire technical skills in a hands-on environment without fear of harming patients, allowing them to increase their technical proficiency in clinical skills, and react to real-life situations with lower stress levels [1]. Simulation training is becoming a staple for medical education; however, its large expense acts as a major barrier to accessibility. A single high-fidelity simulator and its monitoring system can cost upwards of $200 000, excluding replacement parts such as synthetic skin and bodily fluids [1]. Lower-fidelity models are more cost-effective and only require basic technology if any at all, but they are static and lack realism or situational context [1]. That being said, low-fidelity models such as task trainers can be sufficient for technical skill training [2]. 

Rural or remote areas have limited access to simulation labs, resources, and clinical skills instructors, which can be prohibitive to skill maintenance [3]. There is a lack of opportunity to practice “high stakes low-frequency” procedures, such as chest tube insertion, despite an increased rate of injury-related death in rural areas [4]. These healthcare professionals often lack access to advanced skills training or opportunities to attend educational sessions [3]. Herein, we have created a telesimulation delivery model that will bridge the gap between rural and urban access to simulation-based learning. Telesimulation aims to educate, train, and/or assess participants at an off-site location [5]. It is demonstrated to be a cost-effective method of simulation training for participants at less-accessible off-site locations, who are unable to take advantage of similar educational tools in person [5]. By eliminating time and distance barriers in educational content delivery, the opportunity for the rapid circulation of new medical education brings the benefits of simulation training beyond the walls of the centre, while simultaneously creating an inter-institutional network and collaboration opportunity [5]. Without continued practice, technical skills may regress. Simulation of essential skills allows doctors understand current practices on proper insertion of chest tubes. Literature on cost-effective telesimulation models for high stakes, low-frequency clinical procedures is limited. This technical report describes a novel chest tube insertion telesimulation model to add to the growing body of research in the area.

The overall goal of this technical report was to increase the technical performance of learners, including medical students, residents, and doctors located in resource-restricted areas in placing chest tubes. The learning objectives for the case are to clinically diagnose tension pneumothorax, become proficient in the chest tube insertion procedure, develop effective interpersonal and communication skills by calling for appropriate back up/help when needed, and effectively communicate with colleagues. 

## Technical report

The case

A 35-year-old male presents to your rural hospital after striking a moose on the main highway near your town. Paramedics say the truck was found upside down in a ditch and the patient had been ejected from the vehicle. He has multiple injuries including significant bruising of the left chest wall, is pale, and experiencing shortness of breath. He is awake and in pain and is only able to speak about two words per breath. He has neither allergies nor is he on any current medications. His vitals are as follows: heart rate (HR) 120 bpm, O_2_ saturation 93%, and blood pressure (BP) 100/70. Upon examination, the chest sounds are absent on the left side, and the patient’s trachea is deviated to the right. Despite attempted decompression with the appropriate needle thoracostomy, the patient has ongoing respiratory distress and whether the patient requires urgent placement of a chest tube will be determined.

The organization and implementation of all elements of this simulation-based technical report follow a modified Context, Input, Process, and Product (CIPP) program development and evaluation model [6-7]. Its management is closely based on the technical report outlined by Gomersall et al. [8]. Please access the original sources for further details and pictorial representations [6-8].

Context

This simulation can be used in remote, rural, or resource-restricted acute care centers across Newfoundland and Labrador for the distribution of clinical skill training/ refreshers to medical students, residents, doctors, and nurses. These individuals will be connected to an expert skills instructor from the Memorial University’s Clinical Learning and Simulation Centre. The case can be adjusted to accommodate location, learners, modality, and difficulty level to meet the needs of a variety of learners as outlined in Table 1. While there are a number of low-cost chest tube insertion simulations in the literature, there is still minimal research on using telesimulation for instructing chest tube insertion [9-10].

Inputs

Table 1 provides inputs for managing a chest tube insertion procedure for a variety of indications.

**Table 1:** Inputs for managing a chest tube insertion procedure for a variety of indications [4,9-11]

Case Setting
A community hospital where the learner (clerk/resident/doctor) is responsible for leading the case and must call for help if necessary. The learner can obtain backup from a qualified surgeon/physician in their community hospital or seek contact with the simulation instructor through telecommunication. The room setup contains a triage report outlining the case as above and chest tube task trainer consisting of three-dimensional (3D)-printed ribs and skin on a stand affixed to a table.
Personnel
Learners: Medical clerks/Emergency medicine residents/Doctors/Nurses Facilitators: One to two simulation center staff members, one to provide chest tube insertion skills instruction at the Memorial University site, and the other to observe and record information for debriefing technical support: readily available to assist with telecommunication equipment at both locations
Moulage
Minor to severe injuries depending on simulation difficulty - Diaphoretic - Left chest wall bruising - Pale
Supplies
Simulation Equipment
Chest tube task trainer composed of 3D-printed skin and ribs attached to a stand affixed to a table. Alternatively, learners could use an inflated balloon placed under a rack of pork ribs secured to a stand if a 3D-printed model is unavailable.
Medical Equipment
Sterile drapes and gloves - Antiseptic - Local anesthetic (Lidocaine) - Scalpel with No. 10 blade - Chest tube - Large Kelly clamps - Mayo scissors - Gauze, occlusive dressing, tape - Silk suture - Needle driver - Forceps -Suture scissors - Pleur Evac
Technology Equipment
Both sites require the following for two-way audio-visual communication: • Reliable network connections • Computers configured with SKYPE® or similar video calling technology • Microphones and headsets for all participants • At least, one external camera at rural site • Two cameras at Memorial University: a static camera for face-to-face discussion between instructor and trainee, and another camera for hands-free skills demonstration • Dual simulation set-up; so the instructor can demonstrate simulation set-up and skills

Process

The following scenario resembles the case produced by Renouf et al. adapted to fit this chest tube insertion simulation [4]. In this scenario, a nurse acted as a confederate and facilitator of the simulation at the rural site. They initiated contact with the Memorial University instructor prior to the start of the simulation for the purposes of pre-briefing and presenting the case, setting up the simulation equipment, and assisting the physician, who is the learner, during the procedure. If concerned about technical glitches, such as poor connection or difficulty with arranging equipment, the confederate and Memorial University instructor can arrange for extra time before the start of the simulation or at an earlier date to sort these out. Once ready, the physician learner entered the room and the instructor initiated a formal pre-briefing session and presented the case. The role of the instructor is to run the simulation, instruct the learner on the chest tube insertion technique when prompted by the learner, and ensure adherence to the scenario. A second staff member was present at the simulation center to observe and record information for debriefing, as well as assist the instructor during demonstration of the simulated procedure when necessary.

Pre-briefing

During the pre-briefing, the instructor outlined the schedule of events from the start of the simulation to de-briefing. They also emphasized the importance of maintaining a safe learning environment and introduced a quasi-contract. Foreseen technical limitations to telecommunication and simulation can be addressed during pre-briefing to minimize interruptions during the simulation. Learners are briefed on how the formative evaluation is conducted and provided with the initial information about the case.

Scenario

Following the pre-briefing, learners were provided with the pre-scenario information as outlined in Table 2.

**Table 2:** Background case information and expected actions for the chest tube insertion scenario ER, emergency room; HR, heart rate; BP, blood pressure; RR, respiratory rate; SaO_2_, oxygen saturation rate; HEENT, head, eyes, ears, nose, and throat; ABCDE, Airway, Breathing, Circulation, Disability, Exposure/Examination; IV, intravenous

Pre-scenario				
You are working in the ER when a 35-year-old male presents to your hospital after striking a moose on the main highway. Paramedics say the truck was found upside down in the ditch, and the patient had been ejected from the vehicle. He has multiple injuries and is experiencing shortness of breath despite needle thoracostomy conducted by paramedics.				
History (Hx)				
Allergies	None			
Medications	None			
Past Medical Hx	None			
Physical Exam				
Initial Vitals	HR: 120 bpm / BP: 100/70 mmHg / RR: 32 / SaO_2_: 93%			
General	Pale and diaphoretic with significant bruising on the left chest wall, and signs of respiratory distress. The patient is alert, in pain, and having difficulty speaking.			
HEENT	Opens eyes in response to voice, disoriented, obeys commands			
Chest	Heart sounds normal, breath sounds absent on the left. Percussion note is hyper-resonant on the left.			
Abdomen	Soft, non-tender			
Extremities	Open femur shaft fracture ***for increased difficulty variant of the case (learners = doctors or residents)			
Case Progression:				
General Assessment	Vital signs and clinical diagnosis		Expected Action	
Initial assessment 1				
Runs trauma code using the ABCDE approach	Airway (A) - Patent and protected, trachea deviated to right; Breathing (B) - RR 32, respiratory distress, absent breath sounds and increased tympany on left; Circulation (C) - BP 100/70, HR 120, SaO_2_ 93, heart sounds normal, jugular venous pressure (JVP) elevated. Open femur fracture		Identify the need to place the patient on cardiac and oxygen monitors and obtain IV access. Establish the clinical diagnosis of traumatic pneumothorax and identify the need for chest tube insertion. Communicate with nurse confederate to call for back up (either within the hospital or through telecommunication with sim center staff member). Learner may identify need to place addition needle thoracostomy while waiting for chest tube supplies and preparation, the nurse confederate may agree and comment that it has been placed for the purposes of this simulation.	
If DONE within 2 minutes of presentation GO TO CHEST TUBE PREP 1, If NOT DONE within 3 minutes OR if learner FOCUSES ON FEMUR FRACTURE, GO TO INITIAL ASSESSMENT 2				
Initial assessment 2				
The learner is prompted that the patient looks worse Prompt: Nurse comments “Looks like the needle thoracostomy isn’t enough to relieve pressure, perhaps the patient needs a chest tube”		SaO_2_ drops 90		Recognize worsening and/ or need to stabilize breathing before femur injury, immediately calls for backup for chest tube insertion.
GO TO CHEST TUBE PREP 1				
Chest tube prep 1				
Patient appears stable	Vitals stable		Communicate with the nurse and the instructor to obtain equipment and prep patient. Reposition and drape patient and deliver appropriate anesthetics.	
If DONE within 5 minutes go to CHEST WALL DISSECTION 1, if NOT DONE within 5 minutes GO TO CHEST TUBE PREP 2				
Chest tube prep 2				
The learner is prompted that the patient looks worse		SaO2 drops 90, RR: 36		Recognize worsening and works with instructor/nurse in a more efficient manner to obtain equipment and prep patient. Learner may identify the need to place addition needle thoracostomy while waiting for chest tube supplies and preparation, the nurse confederate may agree and comment that it has been placed for the purposes of this simulation.
GO TO CHEST WALL DISSECTION 1				
Chest wall dissection 1				
Patient appears stable	Vitals stable		Identify anatomical landmark for chest tube placement (4^th^/5^th^ intercostal space, mid-axillary line). Make cut and dissect chest wall with Kelly clamp.	
If DONE within 5 minutes after the patient is prepped GO TO CHEST WALL DISSECTION 3, If NOT DONE within 5 minutes after the patient is prepped GO TO CHEST WALL DISSECTION 2				
Chest wall dissection 2				
Prompt - Nurse comments “The patient’s vitals are deteriorating, I think we need to speed it up”	HR rises: 130, SaO_2_ drops 88		Complete incision and blunt dissection with help from backup physician/instructor	
GO TO CHEST WALL DISSECTION 3				
Chest wall dissection 3				
Patient appears stable	Vitals stable		Insert gloved finger into the cavity to ensure positioning in the pleural cavity and clear any adhesions	
If DONE within 30 seconds GO TO CHEST TUBE INSERTION 1, if NOT DONE within 30 seconds GO TO CHEST WALL DISSECTION 4				
Chest wall dissection 4				
Prompt: Nurse comments “Is the site clear of adhesions?”	HR: 130, SaO_2_ drops 85		Insert gloved finger into the cavity to ensure positioning in the pleural cavity and clear any adhesions immediately. May be instructed to do so by backup physician/ instructor.	
GO TO CHEST TUBE INSERTION 1				
Chest tube insertion 1				
Patient appears stable	Vitals stable		Advance correct end of chest tube through cavity using Kelly clamp, ensuring placement directed toward patient’s head, within pleural space and attach Pleur-Evac.	
If DONE within 2 minutes GO TO CHEST TUBE CHEST TUBE PLACEMENT 1, if NOT DONE within 2 minutes OR DONE INCORRECTLY GO TO CHEST TUBE INSERTION 2				
Chest tube insertion 2				
Patient’s condition deteriorating	HR: 130, SaO_2_ drops 83		Recognize chest tube placement error and correct it. May be prompted by backup physician/ instructor on how to do so.	
GO TO CHEST TUBE PLACEMENT 1				
Chest tube placement 1				
Patient’s condition improving		HR decreases 110, SaO_2_ rises 88, RR decrease 22		Secure chest tube in place using suture and dressing, attach to Pleur-Evac. Order X-ray to ensure proper placement.
GO TO END				
End				
Patient’s condition improving, breathing normalizes and can speak to healthcare easier.		Vitals normalize		Simulation complete

Products/Outcomes

The scenario ended when the patient had a functional chest tube inserted and is stable. A feedback and debriefing session occurred next, where all members of the simulation reflected on the learner’s performance, interaction with other members of the trauma team (i.e. nurse and/or backup physician) and the efficacy of the telesimulation model, during which time the learning objectives were reviewed.

Feedback and debriefing

As mentioned previously, all people involved in the simulation participated in a verbal debriefing session following its completion. During this time the instructor and second simulation center staff member discussed the learner’s performance with them for formative purposes on the basis of the learning objectives, emotions, actions, reflection, and next steps (LEARN) framework produced by Memorial University [12]. The teamwork between the learner and assisting nurse was also to be addressed as per the learning objectives. De-briefing provides an opportunity for continued education and sense of completion after a simulation session [13-14]. Time was also taken to discuss the execution of the telesimulation, including any technical issues or instructional difficulties the team might have had, so it can be used in the future to improve on the simulation experience.

An objective structured assessment of technical skills (OSATS) for chest tube insertion was completed by the instructor on learner performance, which will be used as feedback for the learner for continued education and for the simulation center staff to ensure the effectiveness of the telesimulation model. OSATS has been validated as a successful tool in teaching many surgical procedures. The modified OSATS tool that can be found in Table 3 has been adapted from a similar tool created and validated by Friedrich et al. [15-16].

**Table 3:** Objective structured assessment of technical skills tool for evaluation of chest tube insertion based on one created by Friedrich et al. [16]

	1: Poor	2	3: Sufficient	4	5: Excellent
Correct identification of incision location	The chosen dissection plane is not near the suggested site		The chosen dissection plane deviates slightly from the suggested site		4^th^/5^th^ intercostal space, mid/anterior axillary line
Incision on top side of rib	Dissection not performed on top side of rib		Dissection carried out with minor errors		A roughly 2-cm cut is performed on the top side of rib with a clean cut through subcutaneous layers, and intercostal muscle
Blunt dissection of subcutaneous plane	Distance or execution of tunneling lacking		Either distance or execution of tunneling lacking		Confident and accurate execution and distance of dissection into the pleural cavity
Clamp insertion and removal to open path for chest tube insertion	Poor handling of instruments that may cause avoidable harm to the patient. Multiple attempts.		Clamp expanded upon removal, handling could be improved. 1-2 attempts		Confident handling of clamp, smooth removal with clamp expanded to widen the tunnel on the first attempt
Digital exploration of tunnel and pleural cavity to ensure proper position and lack of adhesions between lung and pleural surface	No digital exploration		Finger inserted into pleural cavity, no digital exploration		Thorough digital exploration, with 360^○^ turn, ensure no adhesion of lung to pleural space or blood clots obstructing path
Chest tube insertion into pleural cavity	Hazardous handling of tube that might cause avoidable harm to the patient, no use of clamps Poor tube advancement		Clamp closed on tip of chest tube and used to direct tube into position. Improvable handling, advancement carried out with minor errors		Confident handling with clamp closed on tip of chest tube and used to direct tube into position Clamp removed at appropriate time for manual tube advancement
Tube length and position	Estimated drain length greatly deviates from recommended length, and/or chest tube inserted too far into pleural cavity causing avoidable discomfort		Estimate length or amount of chest tube inserted into pleural space deviates slightly from rater’s opinion		Optimal chest tube length and appropriate insertion into pleural space
Securing chest tube/ suturing	Unsure how to secure chest tube and had significant difficulty performing the anchoring suture or knot Chest tube not secure by rater’s opinion		Room for improvement of suturing skills, but chest tube secure by rater’s opinion		Confident placement of anchoring suture, and securing of chest tube Chest tube secure by rater’s opinion
Length of time required to complete procedure	Many unnecessary or disorganized movements and significant pauses or uncertainty		Some unnecessary movement or nervousness but with organized time and motions		Confident movements with maximum efficiency
Amount of help or assistance needed from tutor	Learner needed multiple demonstrations and much instruction from tutor		Learner was able to complete task follow demonstration with some help from the instructor, only raising important questions to maximize performance.		Learner was able to confidently complete task with almost no assistance from instructor following the initial demonstration
Teamwork between learner and assistant	Poor communication/ execution of team roles		Communication between team members could be improved, but otherwise appropriately carried out roles		Excellent communication between learner and assistant, both members carried out their roles well and in synchrony.
Total Score: __/55					

**Figure 1:**
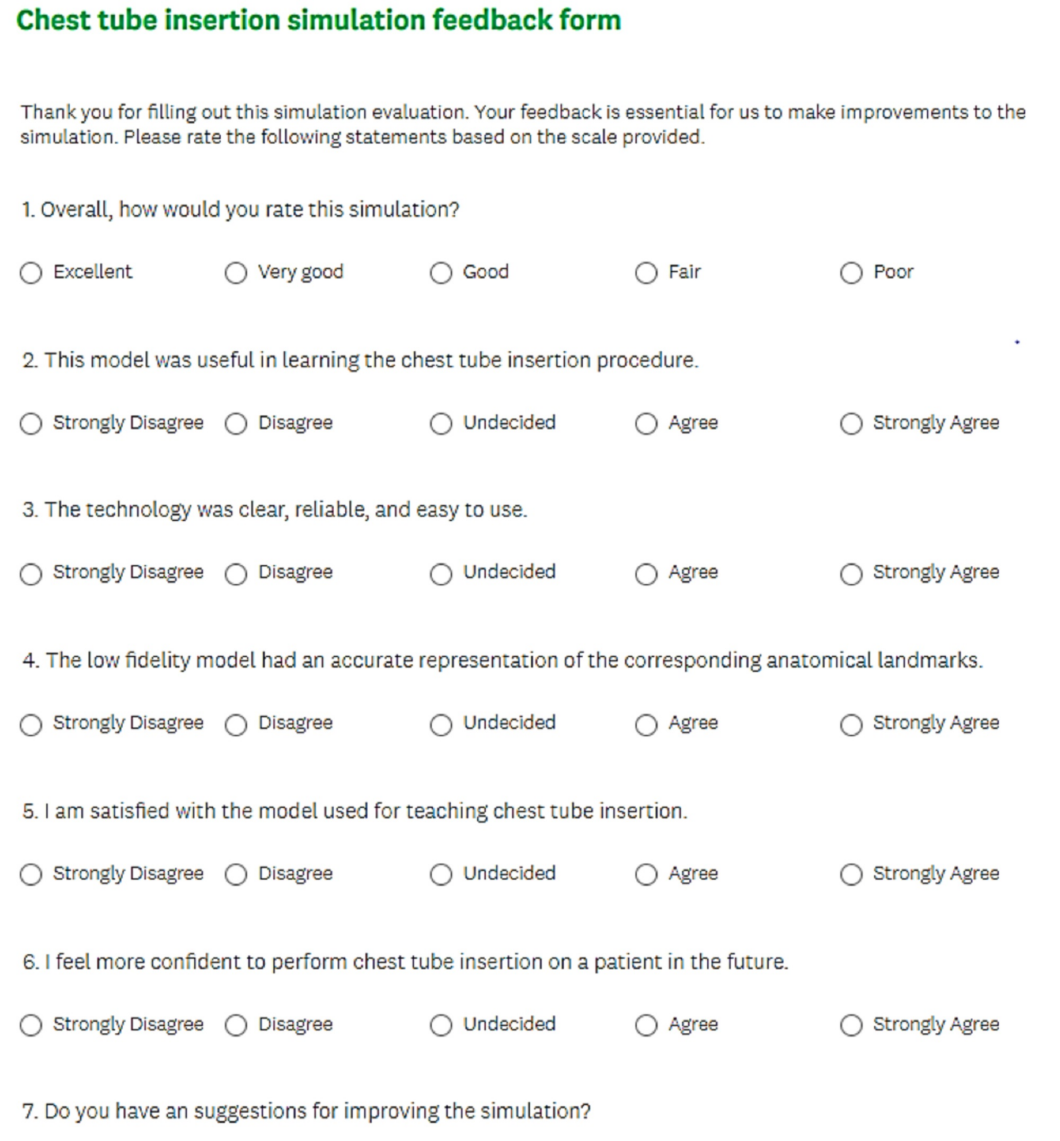
Feedback form for learners to assess learning experience and realism of model

Each trainee will complete a feedback form to evaluate the learning experience and realism of the model as seen in Figure 1.

Participants were asked to rate their opinion of the model on a Likert scale for questions such as the usefulness of the model in learning the procedure, fidelity of the corresponding anatomic landmarks in comparison with clinical reality, degree of self-confidence to perform the procedure on a patient, degree of realism, and satisfaction with the model [17]. 

## Discussion

The use of simulation-based models in the continued education of learners (doctors, residents, and medical students) provides a promising method for ensuring competency in high stakes, low-frequency procedures such as chest tube insertion. Current literature on the use of simulation suggests a relationship between increased standardized learning outcomes and hours of simulation-based medical education [18]. Learners report a greater level of confidence, satisfaction, and enhanced learning regardless of the level of fidelity of the simulation [19]. Waiting for hands-on exposure to low-frequency procedures in a clinical setting is of low yield; therefore, routine simulation-based scenarios are essential for continued competency in these procedures [20].

This simulation scenario offers learners the opportunity to recognize tension pneumothorax and practice the chest tube insertion procedure in a safe, hands-on learning environment. Learner performance and teaching are based on an objective-structured assessment of technical skill tools. Having the learners assemble the supplies needed is a good way to test their knowledge on the location of the required tools and increase efficiency for future emergency situations. It incorporates the use of a nurse confederate to assess interpersonal skills and enhance teamwork, therefore touching on multiple CanMeds competencies. Learners have the option to contact appropriate back-up or help if needed and thus test their knowledge of who and how to contact other professionals in a high-stake setting. Variants in the level of difficulty are provided allowing for adaptability for the level of education of the learner and test their ability to prioritize tasks.

The scenario described in the technical report is of low fidelity because of the added challenge of conducting the scenario over teleconference. It is rather basic because it looks to teach a wide variety of learners. Variants to this scenario may be added, such as tension pneumothorax, femur fraction, or compromised airway, for more advanced learners. In addition, a standardized patient + task trainer variant, as described by Jamie Wilson, may add a dimension of realism, urgency, and patient-centred approach to a chest tube insertion simulation. Future directions for this project would be to test the smoothness of the scenario as it is and determine the feasibility of expanding the telesimulation to a more realistic, higher-fidelity simulation such as the standardized patient + task trainer so that soft skills can be tested and practiced.

## Conclusions

This technical report describes the design and implementation of a low-cost, low-fidelity chest tube insertion simulation using a 3D-printed model for the teaching/practice of physicians, residents, and medical students through telecommunication. The model attempts to bridge the gap in continued education for learners residing in rural and remote areas who cannot access educational sessions and high-fidelity simulators located in urban areas. Low-fidelity simulation provides a safe, controlled learning environment where medical learners can practice their knowledge, procedures, and interpersonal communication without harm to patients. Staying up to date on high-stakes, low-frequency procedures, such as chest tube insertion, is vital to professional practice.

## References

[1] Al-Elq A H (2010). Simulation-based medical teaching and learning. J Family Community Med.

[2] Norman G, Dore K, Grierson L (2012). The minimal relationship between simulation fidelity and transfer of learning. Med Educ.

[3] Glazebrook R M, Harrison S L (2006). Obstacles and solutions to maintenance of advanced procedural skills for rural and remote medical practitioners in Australia. Rural Remote Health.

[4] Renouf T, Parsons M, Francis L, Senoro C, Chriswell C, Saunders R, Hollander C (2017). Emergency management of tension pneumothorax for health professionals on Remote Cat Island Bahamas. Cureus.

[5] McCoy C E, Sayegh J, Alrabah R, Yarris L M (2017). Telesimulation: an innovative tool for health professions education. AEM Educ Train.

[6] Stufflebeam DL, Coryn CLS (2014). Evaluation Theory, Models, and Applications (2nd ed.). https://books.google.ca/books?hl=en&lr=&id=SbnlBQAAQBAJ&oi=fnd&pg=PA133&dq=Stufflebeam+DL,+Shinkfield+AJ:+Evaluation+Theory,+Models,+and+Applications,+2nd+ed.+Jossey-Bass,+San+Francisco,+CA%3B+2014.&ots=ajCvoJQ00Z&sig=WnvbOSlGbcP0sme08i06Vr9Da1k#v=onepage&q=StufflebeamDL%2CShinkfieldAJ%3AEvaluationTheory%2CModels%2CandApplications%2C2nded.Jossey-Bass%2CSanFrancisco%2CCA%3B2014.&f=false.

[7] Dubrowski A, Alani S, Bankovic T, Crowe A, Pollard M (2015). Writing technical reports for simulation in education for health professionals: suggested guidelines. Cureus.

[8] Wiseman J, Snell L (2008). The deteriorating patient: a realistic but “low-tech” simulation of emergency decision-making. Clin Teach.

[9] Naicker TR, Hughes EA, McLeod DT (2012). Validation of a novel resin-porcine thorax model for chest drain insertion training. Clin Med.

[10] Van Doormaal CJ, Howes DW, Salazar CL, Parker CM (2011). 112 An innovative and inexpensive pork ribs model for teaching tube thoracostomy. Ann Emerg Med.

[11] Netto FACS, Sommer CG, Constantino Mde M, Cardoso M, Cipriani RFF, Pereira RA (2016). Teaching project: a low-cost swine model for chest tube insertion training. Rev Col Bras Cir.

[12] (2017). Faculty of Medicine, Memorial University of Newfoundland. Tuckamore simulation research collaborative - The LEARN framework. [online]. http://www.med.mun.ca/TSRC/Cureus/LEARN.aspx.

[13] Sawyer T, Fleegler M B, Eppich W (2016). Comprehensive Healthcare Simulation: Essentials of Debriefing and Feedback.

[14] Fanning R M, & Gaba D M (2007). The role of debriefing in simulation-based learning. Simul Healthc J Soc Simul Healthc.

[15] Friedrich M, Bergdolt C, Haubruck P (2017). App-based serious gaming for training of chest tube insertion: study protocol for a randomized controlled trial. Trials.

[16] Faulkner H, Regehr G, Martin J, Reznick R (1996). Validation of an objective structured assessment of technical skill for surgical residents. Acad Med.

[17] Ghazali A, Breque C, Léger A, Scépi M, Oriot D (2015). Testing of a complete training model for chest tube insertion in traumatic pneumothorax. Simul Healthc J Soc Simul Healthc.

[18] McGaghie WC, Issenberg SB, Petrusa ER, Scalese RJ (2006). Effect of practice on standardised learning outcomes in simulation‐based medical education. Med Educ.

[19] Hoadley TA (2009). Learning advanced cardiac life support: a comparison study of the effects of low-and high-fidelity simulation. Nurs Educ Perspect.

[20] Gordon JA (2012). As accessible as a book on a library shelf: the imperative of routine simulation in modern health care. Chest.

